# A dual-stochastic real options model for sports tourism investment: A case study in China

**DOI:** 10.1371/journal.pone.0339242

**Published:** 2025-12-22

**Authors:** Tao Liu, Jiaxin Cheng, Yong Huang

**Affiliations:** 1 School of Physical Education, Guangdong University of Finance and Economics, Guangzhou, Guangdong, China; 2 School of Physical Education and Health, Guizhou Minzu University, Guiyang, Guizhou, China; University of Almeria: Universidad de Almeria, SPAIN

## Abstract

As the deep integration of sports and tourism continues, sports tourism projects are emerging as an important driver of regional economic transformation and consumption upgrading. However, these projects typically exhibit strong revenue seasonality, long construction cycles, and high market uncertainty, which makes traditional static evaluation methods inadequate for capturing investment feasibility and risk structures. Based on the theory of investment under uncertainty, this study develops a dual-stochastic real options model that couples an Ornstein-Uhlenbeck process with exogenous seasonality (ES-OU) for revenues and a Geometric Brownian Motion (GBM) for construction costs, to characterize the dynamic investment boundary and optimal entry timing under multiple risk constraints. The model is solved using a hybrid numerical framework that combines the Crank–Nicolson–ADI finite-difference scheme with Monte Carlo simulation, ensuring both accuracy and stability. Using the Fanjingshan Mountain Sports Experience Base in Guizhou as a comparable case, parameters are calibrated and simulations conducted with Guiyang’s tourism income and the CSI 300 Building Materials Index. The results show an optimal investment boundary of approximately CNY 196 million per quarter, an investment trigger probability of 62.4%, and a median trigger time of about 3.75 years, indicating a pronounced “delay-and-time-the-entry” decision pattern. Further analysis identifies construction cost and the long-run mean of revenues as the key economic drivers of entry timing: higher costs significantly postpone investment triggers, whereas improved revenue expectations advance entry. These findings validate the feasibility and explanatory power of the real options model in the sports tourism context, reveal the dynamic mechanism of investment behavior under joint revenue–cost uncertainty, and provide quantitative guidance for investment timing, risk identification, and policy design in sports tourism projects.

## Introduction

Since 2019, the Chinese government has introduced a series of policies aimed at promoting the integration of the sports and tourism sectors. Through the implementation of demonstration projects in premium sports tourism, the policy agenda seeks to expand consumption domains such as fitness, sports events, training, and tourism activities, thereby fostering a favorable institutional environment for the growth of the sports tourism industry. The coordinated development of sports and tourism is not only a strategic move to advance the “Healthy China” initiative, but also a practical manifestation of the “Two Mountains” principle of sustainable development. According to data from the United Nations World Tourism Organization (UNWTO), the global sports tourism sector has maintained an average annual growth rate of approximately 15%, whereas China’s sports tourism market has grown at a significantly faster rate of 30–40%, far exceeding the global average [1]. Despite these substantial economic and social benefits, the industry in China still faces structural imbalances on both the supply and demand sides, as well as insufficient integration with market mechanisms [2]. In response, relevant government departments have proposed measures to accelerate the development of the sports tourism consumption market and to continuously improve the supply system, with the goal of further stimulating private-sector investment in this emerging sector.

Enhancing corporate willingness to invest in sports tourism depends fundamentally on the scientific evaluation of economic returns. Since profit maximization is the core objective of enterprise investment, robust economic feasibility assessments not only help attract capital but also play a vital role in optimizing resource allocation, reducing investment risks, and promoting sustainable industrial development [3]. However, investment in sports tourism projects is often subject to multiple layers of risk. On the revenue side, seasonal imbalances in supply and demand cause significant fluctuations in cash flow. In off-peak seasons, falling income can severely erode profitability [4]. On the cost side, the construction phase is exposed to numerous uncertainties, including volatile commodity prices, budget overruns caused by engineering changes, and shortages of skilled labor—factors that often exhibit nonlinear propagation effects [5].

Traditional investment appraisal tools, such as Net Present Value (NPV) and Internal Rate of Return (IRR), exhibit three fundamental limitations when applied to long-term, irreversible tourism projects: (1) linear predictive models fail to capture sudden shifts in market supply and demand; (2) static discounting mechanisms ignore the value of managerial flexibility; and (3) rigid decision frameworks overlook dynamic adaptation to evolving external environments [6]. Therefore, assessing the impact of uncertainty on project profitability and devising rational investment strategies are essential not only for maximizing firm-level investment returns but also for informing public policy and improving resource allocation—thereby advancing the high-quality development of the sports tourism industry.

This study establishes a close logical linkage between theoretical innovation and empirical validation. First, from the perspective of dynamic uncertainty management, it constructs a dual-stochastic real options model that integrates an Ornstein-Uhlenbeck process with exogenous seasonality (ES-OU Process) on the revenue side with a Geometric Brownian Motion (GBM) on the cost side, to depict the revenue volatility and investment timing of sports tourism projects under uncertainty. Then, a representative comparable project is selected as a case study, and based on observed data and policy context, numerical techniques such as the finite-difference method and Monte Carlo simulation are employed for parameter calibration and quantitative solution, thereby verifying the model’s applicability and explanatory power in real investment decisions. Through this “theoretical construction—case application—policy implication” research framework, the study not only enriches the analytical methods for investment decision-making in sports tourism but also provides practical decision-support tools for both public authorities and market participants, facilitating the transition of the sports tourism industry from scale-driven growth toward quality-oriented development.

## Literature review and innovations

### Literature review

#### Research on sports tourism.

Existing research on sports tourism has primarily focused on three main areas: the development of sports tourism resources, the competitiveness of sports tourism destinations, and the sustainability of sports tourism. (1) Resource development. Effective development of sports tourism resources is central to the growth of the industry. Studies have shown that the success of sports tourism projects is closely tied to the strategic use of local resources and the degree of innovation in project design. Zhao and Li. (2024) analyzed the spatial distribution of premium sports tourism projects in China and identified a geographic imbalance, with a higher concentration in the east and south compared to the west and north. Their findings also highlighted the significant influence of regional resource endowments on project development [7]. Similarly, Perić et al. (2023) argued that the business models of sports tourism projects should be tailored to regional characteristics and the diverse preferences of tourists. They emphasized the importance of integrating local cultural features with sporting events to create differentiated tourism products [8]. (2) Competitiveness. Research on the competitiveness of sports tourism destinations has largely focused on quantifying regional advantages. For example, Tian et al. (2023) used the TOPSIS model to evaluate the sports tourism competitiveness of cities in the Guangdong–Hong Kong–Macao Greater Bay Area. Their findings showed that Guangzhou, Shenzhen, and Hong Kong lead in competitiveness, while Macau and inland cities lag behind [2]. In a similar vein, Moradi et al. (2022) assessed the competitiveness of urban clusters and concluded that success in sports tourism is not only determined by resource endowments, but also by government support, infrastructure quality, and event branding. Their study suggests that enhancing regional competitiveness requires cross-sector collaboration and integrated development strategies [9]. (3) Sustainability. As sports tourism projects pursue economic returns, they must also consider environmental and social sustainability. Yang et al. (2025) highlighted the significant impact of climate change on outdoor sports tourism and proposed both mitigation and adaptation strategies, including improvements in infrastructure and environmental protection measures [10]. Dong et al. (2025) further argued that effective management of sports tourism requires not only supportive public policies, but also collaborative partnerships between government and private capital to ensure long-term sustainable development of the sector [11].

As sports tourism projects shift from conceptual development to practical implementation, scientifically informed investment decisions have become a critical factor in ensuring the sustainable growth of the industry. Such projects often involve large-scale destination construction and infrastructure development, characterized by substantial capital requirements and long payback periods [12]. In addition, demand for these projects is highly sensitive to seasonal fluctuations and broader macroeconomic conditions [4,13]. Sports tourism projects are also unique in that they deliver both economic and social benefits—such as employment generation and destination branding—while involving multiple stakeholders, including governments, private enterprises, and local communities [14]. As a result, investment decisions in this sector must strike a balance between short-term financial returns and long-term strategic value creation [15]. At the same time, these projects face numerous sources of uncertainty that can affect both risks and expected returns. Uncertainty in consumer preferences, income levels, and visitor flows can directly impact revenue projections [16], while changes in tax regimes, land use regulations, and industry oversight can significantly alter cost structures and profitability [17,18]. Ignoring such uncertainties may lead to overinvestment or underinvestment, thereby undermining the long-term sustainability and viability of sports tourism initiatives.

#### Research on investment decision methods.

Investment decision methods have evolved from deterministic static evaluations to dynamic approaches under uncertainty and can be broadly categorized into four types.

The first category is the traditional Discounted Cash Flow (DCF) method. Represented by indicators such as NPV, IRR, and payback period, its core idea is to discount future cash flows to their present value using an appropriate discount rate for project comparison. The method is straightforward and practical but relies on the assumptions of stable cash flows, project irreversibility, and static decision-making. For projects with high uncertainty, staged investment options, or delayed entry opportunities, it neglects the flexibility value of “waiting—learning—redeciding,” which may lead to premature investment or underestimation of potential returns [19].

The second category is the risk-adjusted and scenario analysis method. By employing risk-adjusted discount rates, sensitivity analysis, and scenario tree models, it perturbs key variables such as prices, costs, and tax rates to identify sources of risk and threshold conditions [20]. Although these methods are useful for policy evaluation and cost control, they remain static in nature and cannot endogenously determine the optimal timing of investment.

The third category is the probabilistic and numerical simulation method. Decision tree models and Monte Carlo simulations estimate the project’s NPV and trigger probabilities through multi-path sampling under uncertainty, and are widely applied in infrastructure and energy investment analyses [21]. Their strength lies in capturing the distribution of returns and tail risks, but without incorporating dynamic optimal decision rules, they still cannot generate endogenous investment boundaries.

The fourth category is the real options and dynamic stochastic optimization method. This approach treats investment as an option with the right to exercise, and under uncertainty and irreversibility, determines the dynamic boundaries of “entry—delay—abandonment” through optimal stopping theory [22]. Compared with the traditional DCF framework, it explicitly incorporates learning effects and managerial flexibility, enabling the evaluation of staged investments and optimal entry strategies. The modeling framework has evolved from the initial GBM to include mean reversion, seasonality, jump diffusion, and multi-factor settings; numerically, it has advanced from analytical solutions to finite difference, lattice, and least-squares Monte Carlo methods. This approach has been widely adopted in high-uncertainty fields such as energy, infrastructure, and cultural tourism investments [23,24], becoming a mainstream tool for dynamic investment decision-making.

#### Research progress on investment evaluation in sports tourism.

At present, studies applying the real options approach to investment evaluation in the field of sports tourism remain relatively limited. Most existing research focuses on qualitative analysis or employs static evaluation frameworks based on traditional DCF methods. However, with the growing marketization and increasing uncertainty of the tourism industry, the real options analysis has gradually been introduced into tourism investment research to address specific challenges such as demand volatility, policy sensitivity, and the irreversibility of risk.

At the valuation level, Lin et al. (2013) were the first to apply option pricing theory to the valuation of scenic area operation rights, demonstrating that this method more effectively captures market uncertainty and managerial flexibility, providing a more accurate reflection of potential investment value compared with traditional DCF models [25]. Michailidis (2006) further highlighted through case studies that some tourism projects deemed feasible under conventional methods may reveal hidden risks when reassessed within a real options framework, indicating the method’s significant advancement in risk identification and project screening [26]. In terms of investment timing decisions, Guo et al. (2020) developed a tourism investment model incorporating jump-diffusion characteristics, systematically embedding the risks of unexpected events—such as policy adjustments or public health incidents—into the analytical framework. Their empirical results showed that exogenous shocks have a significant impact on the optimal entry timing [27]. Piñeiro-Chousa et al. (2021) combined the real options approach with cost—benefit analysis to construct an integrated evaluation framework that simultaneously considers economic returns and sustainability objectives, offering a quantitative tool for balancing the dynamic relationship among risk, return, and sustainability in tourism investment [28].

#### Literature commentary.

Existing studies on sports tourism development, investment decision-making methods, and the application of real options provide valuable theoretical foundations and insights for this research, yet several significant limitations remain:

1. Theoretical aspect. In tourism research, current studies pay insufficient attention to project-level investment feasibility and the dynamics of risk. Most analyses are still based on static frameworks or single-source uncertainty assumptions. Such overly simplified modeling approaches create structural deviations between theoretical frameworks and the real investment environment, thereby limiting the explanatory power of tourism investment research in terms of risk measurement and timing decisions.
2. Methodological aspect. Although real options theory has been increasingly applied in tourism investment evaluation, most existing studies rely on a single stochastic process, which fails to adequately capture the intrinsic coupling between dual uncertainties in project revenues and costs. Moreover, current algorithms face the “curse of dimensionality” when solving high-dimensional partial differential equations with multiple state variables, constraining the scalability and applicability of models under complex dynamic environments.
3. Policy integration. While prior studies acknowledge policy factors such as tax incentives and land-use regulations, they typically treat these as exogenous parameters, lacking mechanisms to endogenously embed policy variables within dynamic decision-making frameworks. Consequently, research on institutional simulation and policy feedback based on real options remains scarce, and an interactive system linking investment decision-making with policy optimization has yet to be established.

### Research framework and innovations

This study focuses on sports tourism complexes — typical uncertain investment projects characterized by high capital intensity, strong revenue seasonality, and dual constraints from natural conditions and event cycles. It addresses the core question of how to scientifically determine the optimal investment timing and boundary under multiple sources of uncertainty by constructing a systematic real options analysis framework. The main research tasks and innovations are as follows:

1. Methodological innovation. This study is the first to systematically introduce a dual-stochastic real options model into the field of sports tourism investment, realizing dynamic coupling between revenues and costs. The revenue side adopts an ES-OU process component to capture the seasonal fluctuations and long-term equilibrium of sports tourism projects driven by both climatic and event cycles. The cost side employs a GBM process to describe the dynamic evolution of construction costs under macroeconomic and market disturbances. Compared with traditional models that consider only a single stochastic process, this study extends the framework from “single-risk driven” to “dual-risk interaction,” enabling the characterization of investment behavior under cross-scenarios such as “high revenue—high cost” and “low revenue—low cost.” The model thus achieves dynamic linkage between revenue and cost at the theoretical level, improves the precision and robustness of optimal investment timing determination at the methodological level, and enhances the interpretability and applicability of investment boundaries at the policy level, thereby expanding the application scope of real options theory in sports economics and cultural–tourism investment.
2. Technical innovation. In terms of solution methods, this paper proposes a hybrid algorithmic framework that integrates the Crank–Nicolson–ADI finite-difference method with Monte Carlo optimal stopping simulation to solve high-dimensional stochastic control problems. This approach not only accurately depicts the optimal exercise boundary under complex stochastic processes but also verifies the reliability and precision of numerical solutions through CFL stability, grid independence, and convergence tests. Compared with traditional DCF methods, the proposed model emphasizes the timing of investment and dynamic triggering mechanisms, allowing for the quantification of the probability, temporal, and boundary responses of investment triggers under different scenarios—thus providing a more practical dynamic decision-making basis for high-uncertainty projects such as sports tourism.
3. Empirical application innovation. This study integrates a complex theoretical model with localized empirical data for validation. Taking the Fanjingshan Mountain Sports Experience Base in Guizhou Province as a case study, it calibrates parameters and conducts dynamic simulations using Guiyang’s tourism income and the CSI 300 Building Materials Index. The results identify the optimal entry threshold and trigger timing for sports tourism investment, quantify the sensitivity effects of key variables such as cost, discount rate, and tax rate, and establish a transferable quantitative evaluation framework applicable to sports event projects and regional culture–tourism integration initiatives.

**Fig 1:**
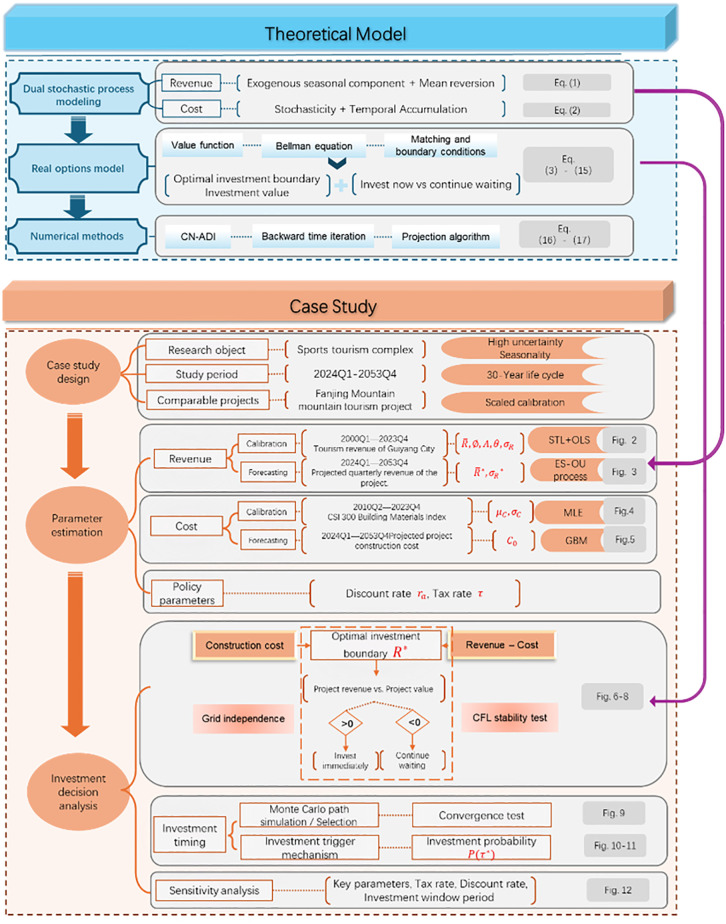
Overall research framework and workflow of the main content.

To aid readers in understanding the overall research logic, Fig 1 presents the general workflow of this study, including the main steps of both theoretical model construction and case study application.

## Variable estimation and model construction

### Stochastic process modeling of project revenue

Compared with general tourism, sports tourism activities exhibit more pronounced and asymmetric seasonal fluctuations. Ordinary tourism demand is mainly affected by holiday schedules and general climatic conditions, resulting in relatively moderate seasonal variations. In contrast, sports tourism is jointly constrained by natural conditions (such as temperature, precipitation, and terrain) and event cycles [29]. For example, activities such as mountain hiking, cross-country cycling, and skiing typically have well-defined “window periods,” during which operations nearly cease entirely when climatic conditions are unfavorable or event schedules are vacant. Consequently, sports tourism revenues tend to display higher peaks, deeper troughs, and asymmetric seasonal dynamics characterized by “rapid rises followed by gradual declines” over time [4].

Although sports tourism revenues fluctuate significantly with climate and event cycles, they are constrained in the long run by fundamentals such as regional economic level, market capacity, facility carrying capacity, and policy support—thus exhibiting an evident mean-reverting pattern. When revenues exceed their long-term equilibrium level, factors such as demand saturation, price ceilings, and diminishing marginal returns cause growth to decelerate and revert. Conversely, when revenues fall below equilibrium, improved weather or the resumption of events quickly restores demand, driving revenues back toward the long-term mean. In contrast, a GBM assumes unbounded stochastic wandering of revenues, lacking a long-term equilibrium constraint, and therefore fails to capture the reversibility and equilibrium properties of sports tourism revenues. Hence, a mean-reverting process better aligns with the economic logic and statistical characteristics of sports tourism income.

Based on these distinctive features, this study introduces an ES-OU to model project revenues. By incorporating an exogenous seasonal term into the traditional mean-reverting framework, the model simultaneously captures both the periodic oscillations and the long-term mean-reversion trends of sports tourism revenues. Its advantages are threefold: (1) it describes the cyclical oscillation patterns of revenues across different seasons; (2) it retains the mean-reversion property, ensuring that revenues fluctuate around a stable level rather than increasing or decreasing without bound; and (3) it effectively distinguishes the differences in volatility and reversion speed between sports tourism projects and general tourism projects [30].

Let R(t) denote the project revenue at the end of each quarter (March, June, September, and December). The dynamic process for revenue is defined as:


$$dR(t)=\theta \left[\overset{―}{R}\left(\text{1}+Asin\left(\frac{\text{2}\pi t}{T}+\emptyset \right)\right)-R(t)\right]dt+\sigma_{R}{dW}_{t}^{R}$$ (1)

Here θ represents the mean reversion rate, reflecting the speed at which revenue returns to its long-term equilibrium; $\overset{―}{R}$ denotes the long-term equilibrium level of project revenue; A and ∅ indicate the amplitude and phase of the seasonal component, describing the periodic variation of revenue within a year; T is the seasonal cycle (one year corresponds to T=4 quarters); $\sigma_{R}$ is the volatility of revenue; ${dW}_{t}^{R}$ is a standard Wiener process.

### Stochastic process modeling of construction costs

Construction costs for sports tourism projects are affected by multiple uncertainties, including commodity price fluctuations, the macroeconomic environment, labor market conditions, and structural supply—demand imbalances. They typically exhibit both randomness and temporal accumulation [31]. To capture these features, this study models construction costs during the investment decision period using a GBM.

The GBM specification has clear economic meaning. First, costs may fluctuate in the short run due to materials prices or macro inflation. Second, in the long run, construction costs show accumulation and irreversibility—once increased, they are difficult to fully revert—reflecting the “stickiness” of capital goods investment. The modeling focus is restricted to the cost evolution before investment occurs [32], i.e., the dynamics faced by investors when deciding whether to enter, rather than post-completion operating or maintenance costs. After completion, related expenditures become fixed capital and no longer affect the determination of the optimal investment boundary.

Let C(t) represent the construction cost at quarter t. The cost dynamics are defined by the following stochastic differential equation:


$$dC(t)=\mu_{C}C(t)dt+\sigma_{C}C(t){dW}_{t}^{C}$$ (2)

Here $\mu_{C}$ is the expected growth rate of construction cost. $\sigma_{C}$ is the volatility of construction cost. ${dW}_{t}^{C}$ is a standard Wiener process associated with construction costs. Revenues and construction costs are assumed to have independent Wiener processes, i.e., $Cov\left({dW}_{t}^{R},{dW}_{t}^{C}\right)=\text{0}$ [22]. This assumption accords with the economic reality of sports tourism projects: revenues are mainly driven by natural and demand factors, whereas construction costs are driven more by macro market and supply factors; the sources differ markedly, implying weak correlation.

### Real options model based on dual stochastic processes

Let V(R,C,t) denote the value of the investment opportunity at quarter t, given revenue R and construction cost C. The investor decides whether to invest immediately or continue waiting. The Bellman equation for the optimal control problem is:


$$\delta V(R,C,t)=max\left\{\underset{The\;Option\;Value\;of\;Waiting}{\underbrace{\frac{\partial V}{\partial t}+LV(R,C,t)}},\underset{The\;Value\;of\;Immediate\;Investment}{\underbrace{\Pi (R)-I(C)}}\right\}$$ (3)

Here δ is the discount rate (with inflation implicitly adjusted), Π(R) is the present value of the post-investment quarterly cash flow (i.e., revenue minus operating costs, discounted), I(C) is the discounted construction cost, and LV(R,C,t) is the infinitesimal generator given by:


$$LV=V_{R}\theta \left[S(t)-R(t)\right]+V_{C}\mu_{C}C+\frac{\text{1}}{\text{2}}V_{RR}\sigma_{R}^{\text{2}}+\frac{\text{1}}{\text{2}}V_{CC}\sigma_{C}^{\text{2}}C^{\text{2}}$$ (4)

with $V_{R}=\frac{\partial V}{\partial R}$, $V_{C}=\frac{\partial V}{\partial C}$, $V_{RR}=\frac{\partial^{\text{2}}V}{\partial R^{\text{2}}}$, and $V_{CC}=\frac{\partial^{\text{2}}V}{\partial C^{\text{2}}}$.

Substituting the generator (4) into the Bellman equation (3) yields the standard PDE:


$$\frac{\partial V}{\partial t}+V_{R}\theta \left[S(t)-R(t)\right]+V_{C}\mu_{C}C+\frac{\text{1}}{\text{2}}V_{RR}\sigma_{R}^{\text{2}}+\frac{\text{1}}{\text{2}}V_{CC}\sigma_{C}^{\text{2}}C^{\text{2}}-\delta V=\text{0}$$ (5)

Let the optimal investment boundary be ${𝛤}^{*}=\left\{R,C,t\right\}$. On ${𝛤}^{*}$, the PDE must satisfy:

1 Value-matching condition: The value of waiting equals the value of immediate investment:

V(R,C,t)=Π(R)−I(C) (6)

2 Smooth-pasting conditions: The first-order partial derivatives of the value function must be continuous at the investment boundary:

$$V_{R}(R,C,t)=\frac{\partial }{\partial R}(\Pi (R)-I(C))$$ (7)

$$V_{C}(R,C,t)=\frac{\partial }{\partial C}(\Pi (R)-I(C))\;$$ (8)

### Numerical solution of the real options model

Since the partial differential equation (Eq 5) depends on both time and two state variables revenue R and construction cost C, it does not admit a closed-form analytical solution. Therefore, this study applies a finite difference method for numerical approximation [33]. The overall procedure includes: time–space discretization, specification of boundary conditions, backward time iteration with projection identification, and numerical implementation with simulation design. The continuous-time Bellman equation is expressed as:


$$\delta V(R,C,t)=\frac{\partial V}{\partial t}+LV(R,C,t)$$ (9)

#### Time and space discretization.

On the time interval [0,T], use quarters as the base unit and further subdivide into sub-steps. The time grid is:


$$t_{n}=n\Delta t,\;n=\text{0},\text{1},\ldots ,N_{t}$$ (10)

In space, construct a uniform grid on the bounded domain $\left[R_{min},R_{max}\right]\times \left[C_{min},C_{max}\right]$:


$$R_{i}=R_{min}+i\Delta R,\;i=\text{0},\ldots ,N_{t};\;C_{j}=C_{min}+j\Delta C,\;j=\text{0},\ldots ,N_{c}$$ (11)

For Eq (9), apply a Crank–Nicolson Alternating Direction Implicit (CN–ADI) scheme in time and central differences in space [34]. For any interior node (i,j), the first- and second-order derivatives are approximated by:


$$V_{R}\approx \frac{V_{i+\text{1},j}-V_{i-\text{1},j}}{\text{2}\Delta R},\;V_{C}\approx \frac{V_{i,j+\text{1}}-V_{i,j-\text{1}}}{\text{2}\Delta C},V_{RR}\approx \frac{V_{i+\text{1},j}-\text{2}V_{i,j}+V_{i-\text{1},j}}{(\Delta R{)}^{\text{2}}},\;V_{CC}\approx \frac{V_{i,j+\text{1}}-\text{2}V_{i,j}+V_{i,j-\text{1}}}{(\Delta C{)}^{\text{2}}}$$ (12)

Substituting the above time and space discretizations into the Bellman Eq (9) yields the discrete system.

#### Boundary conditions.

In practical implementation, the following boundary conditions are applied:

1 Spatial boundaries: If revenue R or cost C exceed predefined limits, the project value is set to 0:

$$V\left(R_{min},C_{j},t_{n}\right)=\text{0},\;V\left(R_{max},C_{j},t_{n}\right)=\text{0},\;V\left(R_{i},C_{min},t_{n}\right)=\text{0},\;V\left(R_{i},C_{max},t_{n}\right)=\text{0}$$ (13)

2 Terminal condition: At the end of the investment horizon T, the option value is assumed to be 0:

V(T,R,C)=0 (14)

3 Immediate investment condition: If the value of immediate investment exceeds the continuation value, then immediate investment is optimal, and the value is updated accordingly:

$$V\left(R_{i},C_{j},t_{n}\right)=max\left\{V\left(R_{i},C_{j},t_{n}\right),\Pi \left(R_{i}\right)-I(C_{j})\right\}$$ (15)

#### Backward time iteration and projection algorithm.

During the solution process, backward time iteration (from  t=T to t=0) is used to obtain the optimal investment boundary. At each time step, the waiting value—the expected return from holding the investment option—is first computed and then compared with the value of immediate investment. If the immediate investment value exceeds the waiting value, the corresponding grid node is identified as the “exercise region.” In numerical implementation, the following projection operator is applied to update the value function:


$$V_{i,j}^{n+\text{1}}=max\left\{V_{i,j}^{n+\text{1}},𝛱\left(R_{i}\right)-I\left(C_{j}\right),\text{0}\right\}$$ (16)

This operator effectively maintains the smoothness and monotonicity of the optimal investment boundary ${𝛤}^{*}$. Through multiple iterations, a convergent optimal investment boundary $R^{*}(C)$ can be obtained.

To ensure stability and convergence, the Courant–Friedrichs–Lewy (CFL) indices of the diffusion and convection terms are computed as follows [35]:


$$\eta_{R}=\frac{\sigma_{R}^{\text{2}}\Delta t}{(\Delta R{)}^{\text{2}}},\;\eta_{C}=\frac{\sigma_{C}^{\text{2}}C^{\text{2}}\Delta t}{(\Delta C{)}^{\text{2}}},\;\xi_{R}=\frac{\theta \Delta t}{\Delta R},\;\xi_{C}=\frac{\mu_{C}C\Delta t}{\Delta C}$$ (17)

According to the stability requirements of the CN–ADI scheme, the parameters Δt, ΔR, and ΔC are selected within the stability region. Subsequently, grid refinement and time-step reduction are performed for convergence testing. By comparing the variations of the optimal investment boundary $\Gamma^{*}$ and the value function V under different resolutions, the insensitivity of the numerical solution to the discretization scale is verified.

#### Computational implementation and simulation environment.

Both the finite-difference solution and the Monte Carlo simulations are implemented on the Python 3.13 platform. Numerical computations use NumPy for linear algebra operations and stochastic process simulation, while SciPy is employed for interpolation and sparse linear system solving. This implementation framework ensures both computational efficiency and reproducibility of the model results.

## Case application and result analysis

### Case selection and data sources

To verify the applicability and robustness of the proposed real options model, this study applies it to the development project of the Guizhou Mountain Sports Complex, a representative investment initiative in China’s sports tourism sector. Guizhou Province is endowed with abundant mountain resources, making it an ideal location for outdoor recreation and sports tourism activities [36]. In recent years, the province has actively promoted the “sports + tourism” development model, supported by strong national and local policies aimed at fostering integration between the two sectors. Selecting this project allows for an exploration of how policy incentives influence investment decisions and project viability, while also showcasing the economic and social benefits of mountain sports infrastructure—such as job creation, local economic growth, and regional development—which are closely aligned with government strategic objectives.

Data used to calibrate model parameters are drawn from several sources. Annual tourism revenue data for Guiyang from 2000 to 2023 were obtained from the Guiyang Municipal Bureau of Statistics. The CSI 300 Building Materials Index, used as a proxy for construction costs, is sourced from the Wind Database. Project-specific information on a comparable case—the Fanjingshan Mountain Sports Experience Base—was obtained from the annual reports of SanTe Cableway Group Co., Ltd.

### Parameter calibration and model simulation

#### Revenue side.

**Parameter calibration:** This study assumes a 30-year project life cycle for the Guizhou Mountain Sports Complex. Due to the absence of historical revenue data specific to the project, quarterly tourism revenue statistics for Guiyang from 2000 to 2023 are used to estimate key revenue parameters.

This setting is justified on two main grounds. First, the model’s core focus lies in the seasonal structure and volatility pattern of revenues rather than the absolute scale of sports tourism income. Therefore, using the seasonal variation of Guiyang’s total tourism revenue as a proxy effectively reflects the cyclical characteristics of the regional tourism market, providing a reliable basis for estimating the parameters of the mean-reversion process with an exogenous seasonal component. Second, official policy documents—namely the Guiyang Sports Tourism Development Plan [37] and the Guizhou Province 14th Five-Year Sports Development Plan [38]—explicitly emphasize that the deep integration of sports events and tourism activities has become a key driver of sustained tourism revenue growth in Guiyang and Guizhou. Large-scale events, such as the Guizhou Mountain Outdoor Sports Conference and the Tour of Guizhou Road Cycling Race, have significantly increased tourist arrivals and overall consumption, confirming the strong positive linkage between sports tourism activities and the broader tourism market. Therefore, approximating the quarterly revenue of the sports tourism project by Guiyang’s total tourism income maintains both data continuity and representativeness, without compromising the accuracy or explanatory power of the model’s estimation of the ES-OU process.

In 2023, Guiyang’s total tourism revenue reached CNY 195.3 billion, with quarterly distributions as follows: Q1: CNY 54.7 billion (28%), Q2: CNY 43.0 billion (22%), Q3: CNY 62.5 billion (32%), Q4: CNY 35.1 billion (18%). The data clearly show that Q1 and Q3 are seasonal revenue peaks, attributable to a combination of holiday effects, favorable weather, cultural events, consumer behavior, and policy interventions. Specifically, the Q1 surge is largely driven by the Chinese New Year period, which, coupled with mild climate conditions and a rich calendar of festivals, significantly boosts tourism demand. The Q3 peak aligns with the summer vacation and Mid-Autumn Festival, during which Guiyang’s cool summer climate positions it as a popular retreat for domestic travelers. In addition, targeted government policies and the development of distinctive tourism products have further stimulated revenue growth in these periods.

**Fig 2:**
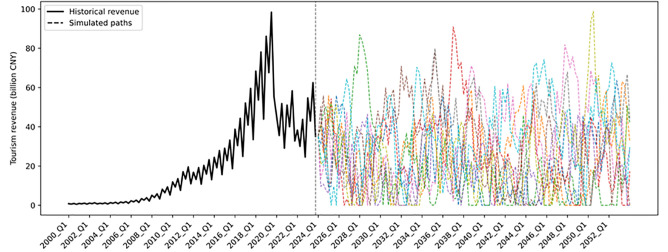
Historical and simulated quarterly tourism revenues of Guiyang.

Based on the 2023 quarterly distribution, a proportional allocation method is used to back-estimate quarterly tourism revenues for 2000–2023, which serve as a proxy to calibrate seasonal revenue patterns for the mountain sports complex. The study employs a combination of Seasonal-Trend Decomposition (STL) and Ordinary Least Squares (OLS) regression to estimate the parameters of the revenue process. STL decomposition extracts the long-term trend and seasonal fluctuations, while OLS estimates the mean-reversion rate and volatility parameters. The results indicate that, under steady-state conditions, the long-term equilibrium level of quarterly tourism revenue in Guiyang is $\overset{―}{R}$≈CNY 21.5733 billion; the seasonal amplitude of fluctuation around this equilibrium is approximately ±11.3%; the seasonal phase is ∅**=**–0.785, implying that the revenue peak occurs in the third quarter, consistent with the typical tourism high season; the mean-reversion rate is θ=0.182, meaning that about 18.2% of deviations from the mean are corrected each quarter, gradually reverting to equilibrium; and the quarterly revenue volatility is $\sigma_{R}$=132.56. Fig 2 presents Guiyang’s historical quarterly tourism revenue from 2000 Q1 to 2023 Q4 and ten simulated revenue paths for 2024 Q1–2053 Q4.

**Quarterly revenue projection:** Based on the ES-OU estimated from Guiyang’s tourism revenue data, this study simulates the future revenue trajectory of the planned Guizhou Mountain Sports Complex. To enhance the credibility of the simulation results, the analysis draws on empirical data from a comparable project—the Fanjingshan Mountain Sports Experience Base—to calibrate revenue expectations.

**Fig 3:**
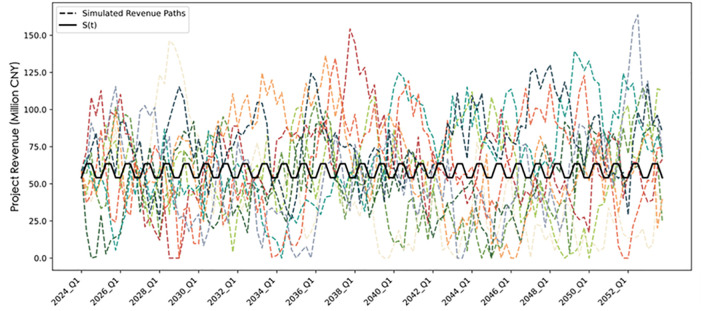
Simulated revenue paths of the project.

The Fanjingshan Mountain Sports Experience Base, located in Jiangkou County, Tongren City, is developed and operated by Sanxiang Cableway Group Co., Ltd. In 2023, the project achieved a total operating revenue of CNY 235 million, with an average quarterly income of approximately CNY 58.8 million. To scale the city-level parameters to the project level, a linear scaling transformation is applied: the ratio of the project’s average quarterly revenue to the city’s long-term equilibrium revenue is used as the scaling factor, $s=\frac{\text{0}.\text{588}}{\text{215}.\text{733}}$. Thus, ${R(t)}^{*}=sR(tRightarrowd{R(t)}^{*}=sdR(t)$, and substituting yields $d{R(t)}^{*}=\theta \left[s\overset{―}{R}\left(\text{1}+Asin\left(\frac{\text{2}\pi t}{T}+\emptyset \right)\right)-sR(t)\right]dt+{s\sigma }_{R}{dW}_{t}^{R}$. This formulation indicates that under the ES-OU, scaling only affects the long-term mean and volatility, while other parameters remain unchanged. Therefore, the project-level parameters are ${\overset{―}{R}}^{*}$=0.588 and ${\sigma_{R}}^{*}$=0.361. Fig 3 presents ten simulated paths of project revenues, showing a clear seasonal mean-reversion pattern consistent with the cyclical nature of sports tourism income.

#### Cost side.

**Parameter calibration:** This study uses the CSI 300 Building Materials Index on a quarterly basis as a proxy for the project’s construction cost to estimate the growth rate ($\mu_{C}$) and volatility ($\sigma_{C}$) of construction costs. The Building Materials Index effectively reflects price fluctuations in construction materials, equipment, and related manufacturing industries, all of which directly influence engineering costs and investment expenditures. Covering core sectors such as steel, cement, and energy chemicals, the index captures the systematic cost variations within the infrastructure construction field and thus serves as a representative and stable proxy indicator for construction costs.

**Fig 4:**
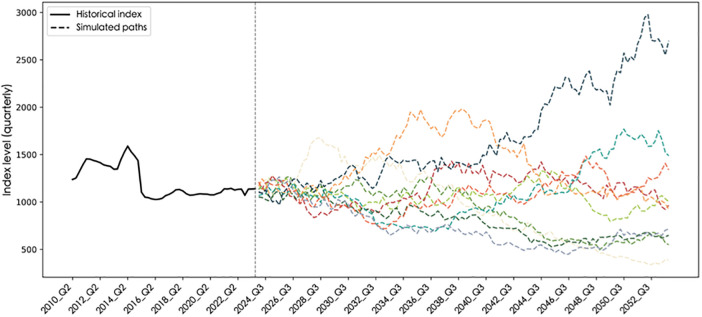
Historical and simulated paths of the CSI 300 building materials index.

The parameters of the GBM process for construction costs are calibrated using the Maximum Likelihood Estimation (MLE) method. The projection assumes that the statistical characteristics of building material prices will remain consistent with historical patterns. Based on the calibrated parameters $\left(\mu_{C},\sigma_{C}\right)$, future cost paths are generated via the Monte Carlo simulation method. The results show a quarterly growth rate of $\mu_{C}$=−0.00055 and a quarterly volatility of $\sigma_{C}$=0.045. This indicates a slight downward trend in building material prices on a quarterly scale, although with noticeable short-term fluctuations. Fig 4 presents the historical data of the CSI 300 Building Materials Index from 2010 Q2 to 2023 Q4 and ten simulated paths for 2024 Q1–2053 Q4, illustrating the dynamic stochastic evolution of construction costs.

**Fig 5:**
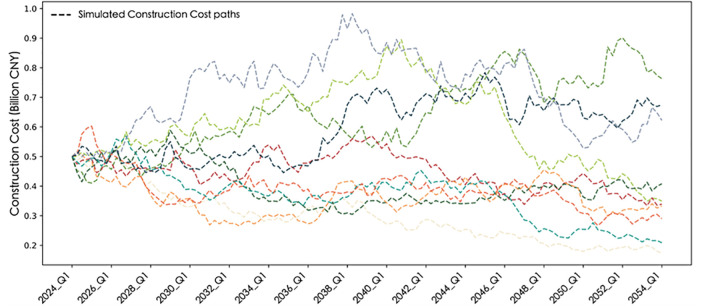
Simulated paths of project construction costs.

**Construction cost projection:** Based on the calibrated parameters of the geometric Brownian motion model, this study adopts the initial investment cost of Fanjingshan Mountain Sports Experience Base ($C_{\text{0}}$=500 million CNY) as the initial value for construction cost of the Guizhou Mountain Sports Complex. Using the GBM framework, the study simulates the evolution of construction cost over the next 30 years, generating 10 stochastic paths to represent potential future scenarios. The simulation results are presented in Fig 5.

### Investment decision solution and result analysis

#### Analysis of the optimal investment boundary.

This study adopts the 2024 one-year Loan Prime Rate (LPR) in China ($r_{a}$=3.1%) as the project’s discount rate. Accordingly, the present value of quarterly revenue R(t) is expressed as: ${PV}_{R(t)}=\frac{R(t)}{{\left(\text{1}+r_{q}\right)}^{t}}$, $r_{q}={\left(\text{1}+r_{a}\right)}^{\text{1}/\text{4}}=\text{0}.\text{0}\text{0}\text{7}\text{6}$. In 2023, the Fanjingshan project reported total operating revenue of CNY 235 million and net profit of CNY 120 million, giving a profit-to-revenue ratio of 1.2/2.35=0.51. The corporate income tax rate for Sanxiang Cableway Group Co., Ltd., the developer and operator of the project, is τ=15%. Therefore, the quarterly after-tax profit of the new sports tourism project is calculated as: $\pi (t)=\frac{R(t)\times \text{5}\text{1}\%\times (\text{1}-\tau )}{{\left(\text{1}+r_{q}\right)}^{t}}$.

**Fig 6:**
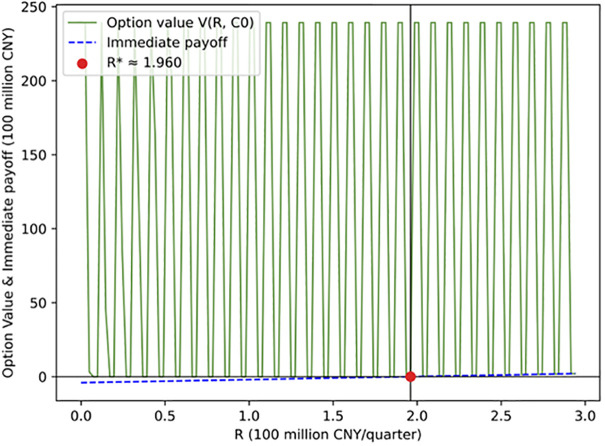
Impact of quarterly project revenue on investment value.

Fig 6 illustrates the relationship between project revenue and investment value under the initial construction cost condition (C(0)=500 million CNY). When project revenue is relatively low (below approximately CNY 196 million per quarter), the immediate investment value is negative (blue line), indicating that investment at this stage would result in a loss; hence, investors should continue to wait. As revenue increases, future project returns rise, and the option value of waiting also increases. When revenue reaches about CNY 196 million per quarter, the two curves become tangent, indicating that the timing for investment is optimal—at this point, the value of immediate investment equals that of continued waiting. Thus, $R^{*}$=1.96 billion CNY per quarter is identified as the optimal investment threshold, implying that when quarterly revenues exceed this level, initiating construction becomes a rational and profitable decision.

**Fig 7:**
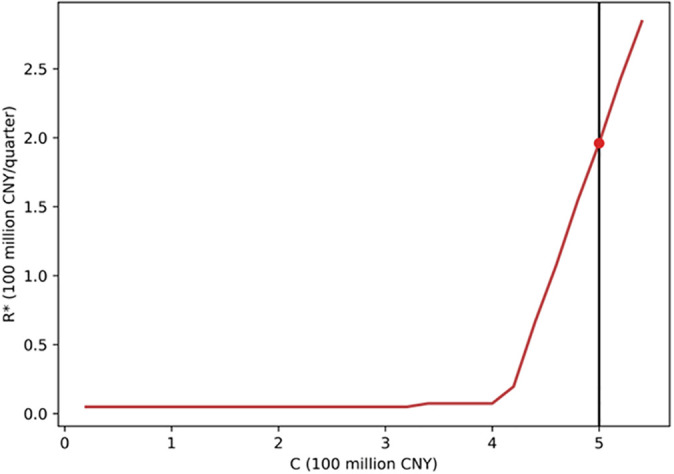
Optimal investment boundaries under different construction cost levels.

Fig 7 illustrates the variation in the optimal critical revenue $R^{*}(C)$ under different levels of construction cost. It can be observed that the investment boundary exhibits a clear positive correlation: the higher the construction cost, the higher the critical revenue required for investment. This indicates that when construction costs rise, firms must wait for higher revenue levels to achieve equivalent investment attractiveness. The result aligns with economic intuition—higher costs and greater risks lead investors to delay investment to avoid potential losses.

**Fig 8:**
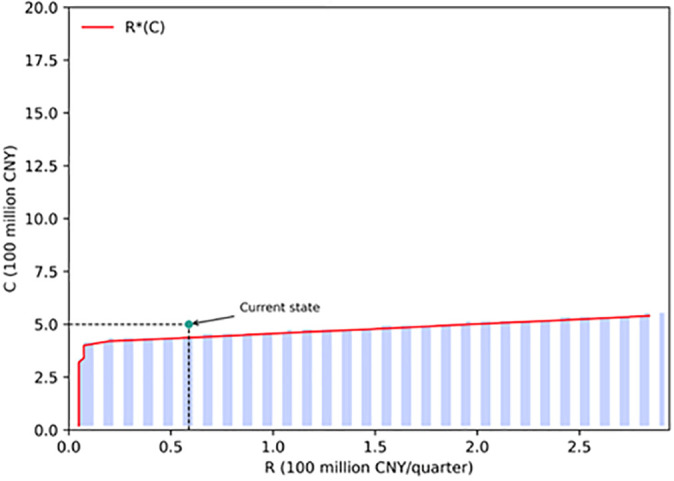
Optimal investment decision regions under revenue–cost combinations.

Fig 8 illustrates the optimal decision regions of investors under different revenue–cost combinations. The purple area represents the immediate investment region, while the white area denotes the waiting region. The red line in between indicates the optimal investment boundary. The current project state (baseline revenue of CNY 58.8 million per quarter and construction cost of CNY 500 million) lies within the waiting region, suggesting that under current conditions, the investment has not yet reached the trigger threshold. Only when revenues increase or construction costs decrease sufficiently for the state point to cross the red line into the purple region does the project become economically feasible for investment.

#### Numerical stability and convergence tests.

Building on the CFL stability conditions and standard convergence requirements, this study sets the baseline discretization as follows: number of grid points in the revenue dimension $N_{R}$=121 and in the cost dimension $N_{C}$=101, with step sizes ΔR=0.0245 and ΔC=0.2; the time dimension uses quarters as the main step, further subdivided into 16 sub-steps, yielding a single-step interval of Δt=0.0625 quarters. The choice of parameters follows three principles: (i) ensure that the CFL and Courant numbers lie within the stability region of the Crank–Nicolson–ADI scheme so that no numerical oscillation occurs under mixed convection–diffusion terms; (ii) based on multiple trials, when ΔR and ΔC are halved, the change in the optimal investment boundary $R^{*}(C)$ is below 3%, indicating convergence; and (iii) keep the overall computation tractable so that the full backward iteration finishes within a few minutes on a standard computer. In sum, the baseline grid and time step achieve a good balance among stability, convergence, and computational efficiency, and can be regarded as the optimal discretization for this model.

To further verify robustness, grid-independence and CFL stability tests are conducted. Expanding the baseline grid from $\left(N_{R},N_{C}\right)$=(121,101) to (182,152), the maximum deviation of the computed optimal boundary $R^{*}(C)$ is only $L_{\infty }$=0.0573, and the relative RMS error is 2.06%, showing that the numerical solution is insensitive to mesh refinement and exhibits good convergence. Meanwhile, the computed diffusion and convection CFL numbers ($\eta_{R}$=6.78, $\eta_{C}^{max}$=0.63, $\xi_{R}^{max}$ =1.12, $\xi_{C}^{max}$=0.0034) all satisfy the stability requirements of the Crank–Nicolson–ADI scheme, confirming that the chosen combination of time and space steps is numerically stable.

### Investment timing result analysis

#### Monte Carlo convergence verification.

To verify the numerical stability and sample adequacy of the Monte Carlo simulation, this study first conducts a convergence test on the estimator of the option value. The number of sample paths is gradually increased N={500,1000,2000,5000,10000,20000}. For each N, ten repeated simulations are performed to compute the sample mean of the estimated option value $\hat{V}$ and its standard error (SE), allowing examination of how SE changes with the number of paths.

**Fig 9:**
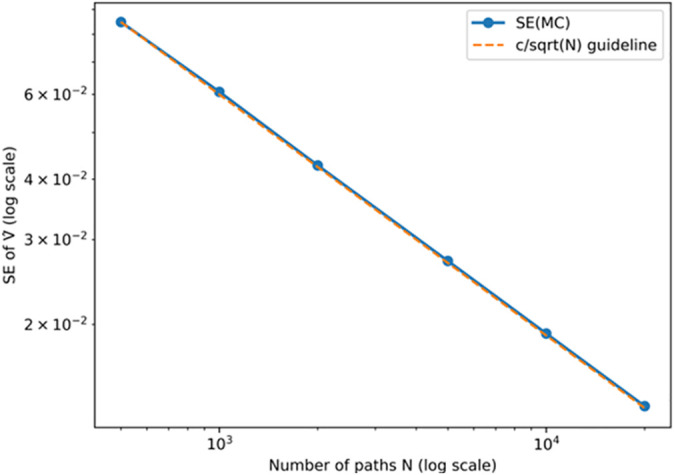
Convergence of Monte Carlo estimation for option value.

Fig 9 shows that as the number of paths increases from 500 to 20,000, the SE of the estimated option value exhibits the typical $\text{1}/\sqrt{N}$ convergence pattern and stabilizes when N>5000. When N=20,000, the results are $\hat{V}$=2.271 and SE = 0.013, with the variance between paths significantly reduced. This indicates that the variance convergence behavior of the model is consistent with theoretical expectations and that the numerical estimates are sufficiently stable. Therefore, the simulation of investment timing in this study adopts a sample size of N=20,000.

#### Investment trigger mechanism and probability distribution analysis.

After verifying the convergence of the Monte Carlo simulation, this study constructs a discrete evaluation and trigger-stability mechanism based on the calibrated optimal investment boundary $R^{*}(C)$ to simulate the dynamic evolution of investment timing. The model assumes that investors evaluate investment opportunities once per quarter. When the quarterly revenue R(t) exceeds the critical boundary value $R^{*}(C)$ for two consecutive quarters, the investment decision is triggered. Based on 20,000 quarterly simulation paths over the period 2024 Q1–2053 Q4, the Monte Carlo experiment shows an investment trigger probability of $P(\tau^{*})$=0.624, indicating that approximately 62.4% of the simulated scenarios result in investment within the simulation horizon.

**Fig 10:**
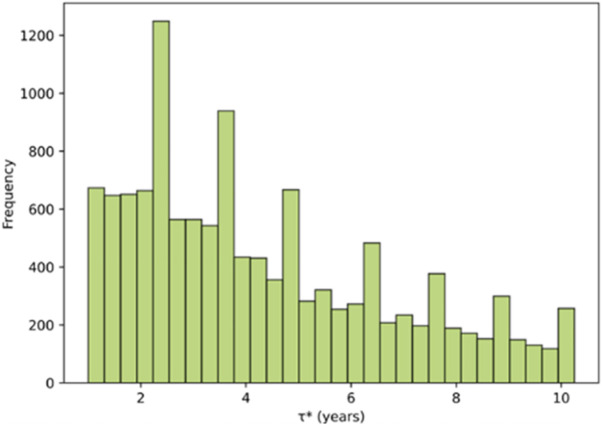
Probability distribution histogram of investment trigger time.

Fig 10 presents the probability distribution of the investment trigger time $\tau^{*}$. The distribution exhibits a distinct unimodal right-skewed pattern, with most triggers occurring between 2 and 6 years, corresponding to the interquartile range of [9,24] quarters (approximately 2026 Q2–2030 Q1). The distribution’s peak lies in the 3–4 year range, aligning with the median trigger time of 3.75 years (2027 Q4), suggesting that the project is most likely to commence around the fourth year after the evaluation baseline. As time progresses, the trigger probability gradually declines; the right-tail of the distribution shows that in a small portion of simulated cases, investment is delayed or not triggered at all. This pattern reflects the temporal dispersion and decision heterogeneity of investment behavior for sports tourism projects under dual uncertainties of market volatility and policy fluctuation.

### Robustness analysis of the investment opportunity value

To verify the statistical robustness of the estimated investment opportunity value, a Monte Carlo convergence test is conducted. Specifically, conditional on the optimal investment boundary $R^{*}(C)$, each simulation round generates 20,000 stochastic paths for revenues and costs, and the experiment is independently repeated 200 times to assess stability under repeated sampling. The results show that the mean investment opportunity value across 200 repetitions is 2.264, with a SE of only 0.0009, yielding a 95% confidence interval of [2.263, 2.266]. This indicates that the estimate exhibits negligible shifts across different random samples and demonstrates strong statistical convergence.

**Fig 11:**
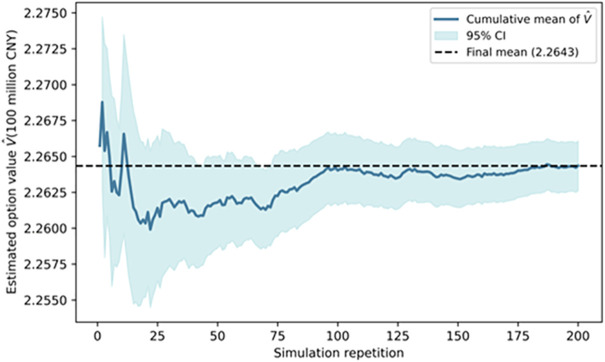
Monte Carlo convergence and confidence interval for the investment opportunity value.

Fig 11 depicts the evolution of the cumulative mean and its 95% confidence interval as the number of repetitions increases. As the repetitions grow from 1 to about 100, the estimate stabilizes rapidly and the confidence band contracts to a very narrow range; additional repetitions thereafter produce only minimal adjustments.

### Sensitivity analysis

**Fig 12:**
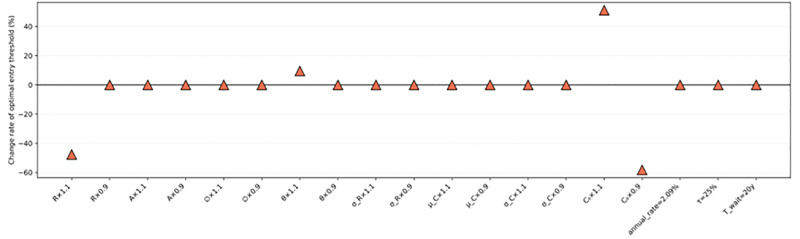
Sensitivity analysis results.

To test the robustness of the model under changes in key parameters, this study conducts a systematic sensitivity analysis focusing on the relative variation of the optimal investment boundary $R^{*}(C)$. The analysis includes four categories of perturbations, with Fig 12 illustrating the percentage change in $R^{*}(C)$ under different scenarios.

1. Core parameter perturbations. The main model parameters ($\overset{―}{R},A,\emptyset ,\theta ,\sigma_{R},\mu_{C},\sigma_{C},C_{\text{0}}$) are each perturbed by ±10% to assess how changes in the revenue structure, cost dynamics, and volatility affect the investment threshold.
2. Tax rate adjustment. In the baseline model, the corporate income tax rate of 15% is based on the preferential policy of the *“*Western Development Program.” However, in practice, since Sanxiang Cableway Group Co., Ltd. did not meet the requirement that 70% of revenue come from its main business, it must repay the preferential tax and penalty. To better reflect the fiscal environment of the Guizhou Mountain Sports Complex, the sensitivity test re-evaluates the model under the standard tax rate of 25%.
3. Discount rate variation. To examine a more conservative benchmark for the cost of capital, the 10-year government bond yield (2.09%) is used to replace the baseline discount rate $r_{a}$=3.1%, evaluating how a lower risk-free rate affects investment assessment.
4. Extension of the investment horizon. To analyze how the flexibility window influences investment timing, the effective project life is extended from 30 to 40 years, examining how a longer investment horizon affects the entry boundary.

Sensitivity analysis results:

1. The long-term mean revenue ($\overset{―}{R}$) has the most significant impact on the optimal boundary. A 10% increase in $\overset{―}{R}$ reduces the critical threshold by approximately 47.62%, indicating a nonlinear response: higher revenue expectations substantially lower the investment barrier and promote earlier entry.
2. When the mean reversion rate (θ) rises by 10%, $R^{*}(C)$ increases by about 15%, implying that with faster revenue correction and market recovery, investors prefer to delay entry and wait for more favorable timing.
3. The initial construction cost ($C_{\text{0}}$) exerts the strongest influence: a 10% increase in $C_{\text{0}}$ raises $R^{*}(C)$ by 58.67%, while a 10% decrease reduces it by 56.12%. This demonstrates that cost factors play a decisive role—any rise in construction costs significantly raises the investment entry threshold.
4. Other parameters ($A,\emptyset ,\sigma_{R},\mu_{C},\sigma_{C}$), as well as variations in the tax rate, discount rate, and project lifetime, have minimal effects, indicating that the model remains stable against short-term and macro-level fluctuations.

Overall, the sensitivity results show that the optimal entry decision is most sensitive to revenue-side structural parameters and the initial construction cost, confirming the model’s ability to capture the key economic mechanisms underlying investment behavior in sports tourism projects.

## Conclusion and implications

### Key findings and discussion

#### Research conclusions.

This study develops a dual-stochastic real options model combining an ES-OU revenue process and a GBM cost process to capture the uncertainty of sports tourism investment. Using the Fanjingshan Mountain Sports Experience Base as a comparable case, the model is solved through finite difference and Monte Carlo simulation methods to determine the optimal investment boundary and timing.

1. Optimal investment boundary and its implications. The numerical solution identifies an optimal threshold of $R^{*}$=1.96 billion CNY per quarter. This means that when quarterly revenue exceeds this level, the net present value of immediate investment equals the option value of waiting, making investment the rational choice. The dynamic balance between revenue growth and construction cost forms the core mechanism of investment decision-making: when revenues are insufficient to offset potential risks and costs, a waiting strategy preserves decision flexibility; as revenues increase or costs decline, the investment boundary gradually converges toward the feasible region, demonstrating the “delay-and-wait” and “timing-to-enter” characteristics central to the real options framework. Further analysis shows that the optimal investment boundary $R^{*}(C)$ increases with higher construction costs, indicating a stable positive correlation between the two. In other words, as costs rise, investors must wait for higher revenues to maintain the same investment attractiveness, which delays project initiation. This outcome is consistent with economic logic. Moreover, the model’s numerical stability and convergence tests confirm the reliability of the results, with parameter settings achieving a sound balance between accuracy and computational efficiency.
2. Investment timing exhibits distinct option-like features. Based on the calibrated optimal boundary $R^{*}$, Monte Carlo simulations reveal the dynamic characteristics of investment triggering under long-term uncertainty. The results show an investment trigger probability of $P(\tau^{*})$=0.624—that is, approximately 62.4% of simulated cases result in investment within a 30-year horizon, suggesting a moderate level of feasibility. The trigger-time distribution exhibits a unimodal right-skewed pattern, concentrated between 2 and 6 years after project evaluation, with a median trigger time of 3.75 years (around 2027 Q4). This implies that investors typically undergo a rational decision process of “wait–observe–enter,” implementing investment once market revenues rise and construction costs stabilize. Economically, the concentration of trigger probability and timing indicates that even under policy and market uncertainty, sports tourism projects remain highly attractive. The decision behavior reflects a typical delay and selectivity effect. Most investments occur within 3–4 years, coinciding with the intersection of high-revenue seasons and cost-decline phases, forming an optimal “high return–low cost” entry window. At this stage, policy and market uncertainties are partially resolved, risk expectations decline, and investment triggers become more concentrated. The right-tail distribution of delayed cases reflects the heterogeneity of investment timing under elevated risk or policy fluctuations.
3. Sensitivity analysis validates the model’s robustness and identifies key economic drivers of sports tourism investment decisions. On the revenue side, an increase in the long-term mean revenue $\overset{―}{R}$ significantly lowers the optimal threshold, indicating that improved revenue expectations encourage earlier entry. In contrast, a higher mean reversion rate θ raises $R^{*}$, implying that when market recovery accelerates, investors tend to delay investment to await a more favorable timing. On the cost side, the initial construction cost $C_{\text{0}}$ exerts the most substantial influence: higher $C_{\text{0}}$ sharply raises the investment threshold, while lower $C_{\text{0}}$ significantly promotes earlier investment. Overall, the model is most sensitive to the structural parameters of the revenue process and the initial construction cost, while showing robustness to macro-level factors such as tax rate, discount rate, and project lifetime. These findings confirm that the model effectively captures the core economic mechanisms governing investment behavior under the dual constraints of revenue and cost in sports tourism projects.

#### Analysis of model generalization.

The dual-stochastic real options model developed in this study breaks through the limitations of traditional static decision frameworks, providing a quantitative analytical tool for uncertainty in sports tourism investment. By jointly considering the seasonality of revenues and the uncertainty of costs, the model is not only applicable to the Guizhou Mountain Sports Complex, but can also be extended to diverse types and regions of sports tourism projects, offering decision support for investment timing and risk assessment.

1. Applicability to urban sports tourism projects. The dual-stochastic model can be equally applied to urban sports tourism projects such as comprehensive sports parks, recreational business complexes, or large-scale event venues. Compared with mountain projects, urban projects exhibit smaller revenue fluctuations but remain influenced by event frequency and holiday effects. In such cases, the seasonal amplitude A is smaller and the mean reversion rate θ is higher, indicating that revenues revert to equilibrium faster and investment timing depends more on short-term changes in policy incentives or consumption demand. Moreover, urban projects typically feature higher capital liquidity and diversified income structures (e.g., ticket sales, venue rentals, sponsorships). To capture this heterogeneity, the model can be extended to include composite revenue terms or multi-factor stochastic processes, thereby improving its descriptive precision.
2. Parameter adjustments under different business models. Sports tourism projects operate under varied business models, each influencing model parameters and investment logic differently: (i) Government-led model: Policy subsidies and land concessions significantly reduce the initial construction cost $C_{\text{0}}$, thereby lowering the investment boundary $R^{*}(C)$ and advancing the investment timing. (ii) Enterprise-led model: With higher market risk and financing costs, both cost volatility ($\sigma_{C}$) and revenue volatility ($\sigma_{R}$) increase, leading investors to adopt a more cautious stance. Consequently, the expected trigger probability $P(\tau^{*})$ decreases. (iii) Public–Private Partnership model: Combining policy stability with market flexibility, this model allows the introduction of a risk-sharing parameter, which flattens the investment boundary $R^{*}(C)$ and widens the trigger region, reflecting the institutional feature of shared risk and joint participation.
3. Regional and sectoral extensions. The model can also be extended to various regional and industrial contexts. For coastal sports and leisure, ice and snow tourism, or event-driven economies, the phase shift (φ) and amplitude (A) of the exogenous seasonal term can be recalibrated based on climatic and activity cycles. For western eco-adventure or health-oriented sports projects characterized by longer payback horizons and greater policy stability, parameters such as the mean reversion rate (θ) and discount rate ($r_{a}$) can be appropriately reduced.

### Research implications

#### Practical implications.

The results indicate that investment decisions in sports tourism projects exhibit pronounced option-like characteristics, where investment behavior follows a pattern of “dynamic entry” rather than a one-time, static commitment. Therefore, enterprises should focus on the following key aspects when planning and managing such investments:

1. Optimizing investment timing decisions. Enterprises should adopt a real options perspective, maintaining flexibility when project revenues have not yet fully materialized and construction costs remain high. When market revenues approach or exceed the optimal entry threshold, firms should move swiftly to capitalize on the favorable investment window. For sports event–driven or culture–tourism integrated projects, early strategic cooperation in event hosting, cross-season operations, and the introduction of experience-based products can help smooth seasonal fluctuations, shorten the waiting period, and improve revenue stability.
2. Strengthening cost control and risk-hedging mechanisms. The study finds that construction cost is the primary constraint influencing investment timing. Firms should mitigate cost volatility in early stages through efficient project design, centralized procurement, long-term supply contracts, and the use of financial derivatives such as building-material futures. Additionally, phased investment or leasing-based financing structures can diversify single-phase risks and enhance capital utilization efficiency, ensuring resilient financial management in uncertain environments.
3. Enhancing information responsiveness and dynamic evaluation capability. Since project revenues and costs are significantly affected by seasonal and policy fluctuations, enterprises should develop data-driven monitoring systems that integrate big data analytics and predictive modeling to dynamically update and recalibrate revenue and cost parameters. By adjusting the investment boundary and project plan in real time, firms can maintain foresight and adaptability amid policy shifts, market volatility, or changing event schedules—ultimately forming a data-informed, adaptive decision-making mechanism for dynamic investment management.

#### Policy implications.

From the perspective of policymakers, the findings of this study provide a theoretical and empirical basis for improving the institutional design of sports tourism investment:

1. Cost reduction is the primary pathway to stimulate investment. Construction cost exerts a strongly nonlinear influence on investment timing—even a small decline in cost can substantially lower the investment threshold and advance the entry point. Governments should therefore reduce initial construction costs through fiscal subsidies, tax exemptions, special-purpose credit programs, and infrastructure support, thereby unlocking investment potential and enhancing the attractiveness of sports tourism projects to private and social capital.
2. Stabilizing returns and strengthening expectations are key to building investor confidence. The long-term mean of revenue has an approximately linear effect on the critical income threshold, indicating that stable revenue expectations can significantly boost investor confidence. Policymakers should strengthen infrastructure development and event scheduling coordination within regional tourism plans to form an integrated “festival–event–consumption” mechanism that mitigates seasonal volatility. In parallel, establishing medium- and long-term policy signaling and information disclosure mechanisms can reduce uncertainty and improve the predictability of market expectations and investment decisions.
3. Rational discounting and risk-hedging mechanisms are equally essential. The results show that a lower discount rate does not necessarily promote investment; rather, it may delay investment by increasing the value of waiting. Hence, interest rate policies and fiscal subsidy timing should be coordinated with risk management instruments. In a low-interest environment, measures such as minimum-return guarantees or income floor mechanisms can reduce investor hesitation and stabilize investment activity.
4. Institutional and environmental support should promote early project implementation. Given fixed discount and revenue conditions, governments can lower the “value of waiting” by improving administrative approval efficiency, providing policy-backed guarantees, and introducing risk-sharing partnerships with social capital. In less-developed regions, it is particularly important to establish a “policy incentive–capital input–market feedback” virtuous cycle, enhancing both the sustainability of sports tourism investments and their regional spillover effects.

### Limitations and future research directions

Although this study offers methodological and computational innovations, several limitations remain. First, the model does not explicitly incorporate project financing structures or capital constraints. Future research could extend the framework to include debt financing, government participation, or mixed-ownership mechanisms, thereby enhancing its explanatory power and practical relevance. Second, the analysis employs static parameter settings and does not dynamically account for long-term variations in policy, market, or climate conditions. Future work could apply multi-scenario simulations or Bayesian updating approaches to improve the model’s adaptability to evolving environments. Finally, the empirical analysis focuses on a single region and project type. Subsequent studies could expand to multiple regions and diverse categories of sports tourism projects to test the model’s applicability and robustness under varying locational, resource, and policy conditions.
